# Analysis of the nursing effects of traditional Chinese medicine featured care combined with individualized care on patients with upper limb fractures

**DOI:** 10.1097/MD.0000000000042772

**Published:** 2025-09-19

**Authors:** Ying Ma, Shuman Qian

**Affiliations:** a Department of Pain Hand Surgery, Wuhan Fourth Hospital, Wuhan, Hubei, China.

**Keywords:** individualized care, negative emotions, quality of life, traditional Chinese medicine featured care, upper limb fracture, upper limb function

## Abstract

This study investigates the nursing effects of traditional Chinese medicine (TCM)-based care combined with individualized care for patients with upper limb fractures. A total of 180 patients with upper limb fractures who received treatment at the hospital’s outpatient department from September 2020 to September 2023 were selected for this study. Through a retrospective analysis of their clinical data, the patients were divided into 2 groups: a control group and an observation group, with 90 cases in each group. The control group received routine care, while the observation group received a combination of TCM-based care and individualized care in addition to routine care. Differences between the 2 groups were compared in terms of symptom recovery time, upper limb function, negative emotions, and quality of life. After nursing care, patients in the observation group showed significantly faster regression of swelling, greater pain relief, and shorter hospital stays compared to those in the control group, with statistically significant differences (*P* < .05). The Fugl-Meyer Assessment Upper Extremity scores of the observation group were significantly higher, while their Hamilton Anxiety Scale scores were significantly lower compared to the control group. Additionally, the Short Form Health Survey scores of the observation group were significantly higher than those of the control group, with all differences reaching statistical significance (*P* < .05).The results of this study suggest that TCM-based care combined with individualized care can effectively shorten the symptom recovery time for patients with upper limb fractures, improve psychological wellbeing, enhance upper limb function recovery, and significantly improve quality of life.

## 1. Introduction

Upper limb fractures are common diseases in clinical orthopedics, often caused by external forces such as falls or impacts, resulting in interruption of the integrity or continuity of the upper limb bones in patients.^[1–5]^ After experiencing a fracture, patients not only suffer from physical pain but may also develop negative emotions such as anxiety and depression due to long-term immobilization and pain, seriously affecting their psychological health and quality of life.^[6,7]^ Therefore, for the nursing care of patients with upper limb fractures, in addition to addressing their physiological symptoms, attention should also be paid to the patients’ psychological and social needs.

In recent years, with the shift in medical models and the updating of nursing concepts, TCM featured care and individualized care have gradually been widely used in clinical nursing.^[8–10]^ TCM featured care, guided by traditional Chinese medicine (TCM) theory, provides personalized nursing plans for patients through the 4 diagnostic methods of inspection, auscultation and olfaction, inquiry, and palpation, aiming to regulate the balance of yin and yang in the patient’s body and promote disease recovery.^[11]^ Individualized care emphasizes the formulation of targeted nursing measures based on the specific conditions and needs of patients to improve patient satisfaction and nursing effectiveness.^[12]^

Currently, research has confirmed the positive roles of TCM featured care and individualized care in the nursing of fracture patients, but there are relatively few studies on their combined application in patients with upper limb fractures.^[13,14]^ Therefore, this study aims to explore in depth the nursing effects of TCM featured care combined with individualized care on patients with upper limb fractures, in order to provide more effective nursing plans for clinical practice, promote patient recovery, and improve quality of life. By comparing and analyzing the differences in symptom recovery time, upper limb function, negative emotions, and quality of life between patients receiving routine care and those receiving TCM featured care combined with individualized care, this study aims to reveal the advantages of the combined nursing model in patients with upper limb fractures. Through this study, we hope to provide theoretical basis for clinical practice and further promote the application and development of TCM featured care and individualized care in orthopedic nursing.

The research hypotheses for the study analyzing the effects of TCM featured care combined with individualized care on patients with upper limb fractures are as follows: The primary hypothesis is that patients with upper limb fractures who receive a combination of TCM featured care and individualized care will have significantly shorter symptom recovery times, including reduced periods for swelling regression, pain relief, and hospital stays, compared to those who receive only routine care. The hypotheses aim to test the potential advantages of TCM featured care and individualized care in improving recovery time, functional recovery, psychological status, and quality of life for patients with upper limb fractures. The validation of these hypotheses is intended to provide more effective nursing plans for clinical practice and to promote the overall recovery of patients.

## 2. Materials and methods

### 2.1. General information

The sample size for this study was determined using a priori power analysis to ensure adequate statistical power. We aimed for a power of 80% (1−*β* = 0.8) to detect a medium effect size, which is commonly used in clinical studies. The effect size was estimated based on a pilot study and previous literature, suggesting that the combined TCM featured care and individualized care could result in a standardized mean difference of ≥0.5 in outcomes such as symptom recovery time, upper limb function, negative emotions, and quality of life. Using G*Power software (Heinrich Heine University Düsseldorf, Düsseldorf, North Rhine-Westphalia , Germany), we conducted power analyses for each of the primary and secondary outcomes. For the primary outcome of symptom recovery time, we set the significance level (*α*) at 0.05, the desired power at 0.80, and the effect size at 0.5. The software indicated that a sample size of 88 patients per group would be required to detect a medium effect size with the specified power and significance level. Considering potential dropouts and loss to follow-up, we inflated the sample size by 10%. Therefore, we aimed to recruit 90 patients for each group, resulting in a total sample size of 180 patients for the study.

In this study, a total of 180 patients with upper limb fractures admitted to the hospital outpatient department from September 2020 to September 2023 were selected as research subjects. The clinical data of the patients were retrospectively analyzed, and based on different nursing methods, they were divided into control group and observation group, with 90 cases in each group. All patients in this study signed informed consent forms, and the study was approved by the Medical Ethics Committee of Danyuan Traditional Chinese Medicine Hospital.

### 2.2. Inclusion and exclusion criteria

Inclusion criteria: patients diagnosed with upper limb fractures confirmed by X-ray examination; local functional limitations, limb pain, swelling, and fracture displacement time greater than or equal to 3 hours; closed fractures, and have undergone internal fixation surgery; complete medical records without missing information.

Exclusion criteria: patients with open fractures or pathological fractures; patients with infections at the fracture site; patients with contraindications to surgery, such as severe cardiovascular diseases, respiratory system diseases, etc; patients with coagulation disorders, organ failure, or other serious diseases; patients with psychiatric disorders or communication barriers.

### 2.3. Methods

Control group nursing: Patients in the control group received routine care. After admission, we provided detailed health education covering surgical methods, possible postoperative complications, etc. At the same time, we explained the perioperative precautions to the patients to ensure that they fully understood the entire treatment process. After surgery, we strictly followed nursing standards for basic and daily care, closely monitored the patients’ recovery, and checked for abnormal conditions such as bleeding or exudation at the surgical incision. In addition, we guided the patients in functional exercises according to the doctor’s orders and provided timely pain relief medication to alleviate their pain.

Observation group nursing: Patients in the observation group received TCM featured care combined with individualized care on the basis of the control group.

#### 2.3.1. Individualized care

We established a dedicated individualized care team, composed of orthopedic surgeons, nutritionists, rehabilitation therapists, orthopedic nursing supervisors, and nurses. The nursing supervisor was responsible for the daily work arrangements and management of the team, and organized regular professional training for the members, inviting orthopedic surgeons to serve as lecturers, focusing on the latest nursing knowledge of upper limb fractures, to ensure that each member could proficiently master it. After the training, we conducted strict assessments to ensure the effectiveness of the training. In the process of formulating individualized nursing plans, we fully considered the patients’ actual conditions, including age, gender, severity of illness, etc. We provided personalized health education and psychological care to the patients, actively communicated with them to understand their feelings and needs, and helped them establish confidence in treatment. For patients with different educational levels, we adopted different educational methods to ensure that patients could fully understand and master relevant knowledge. In terms of surgical cooperation, we strengthened the monitoring of the patients’ vital signs to ensure the safety of the surgical process. The operating room nurse accompanied the patient throughout the process, providing a warm environment to avoid patient discomfort due to low temperatures. At the same time, we also heated the infused fluids and placed a cushion under the patient’s sacrum to improve their comfort. For pain management in patients, we adopted personalized pain care plans. We carefully asked about the patients’ pain sensations and conducted assessments. For patients sensitive to pain, we administered analgesic drugs according to the doctor’s orders; for patients with mild pain, we used other methods such as psychological counseling and physical therapy to alleviate their pain.

#### 2.3.2. TCM featured care

In terms of TCM featured care, we combined TCM theory with the patients’ specific conditions to provide personalized TCM nursing plans. This included using techniques such as Chinese massage and manipulation to promote blood circulation in the patient’s upper limbs, relieve muscle tension and pain; prescribing Chinese herbal medicine formulations according to the patient’s constitution and condition for regulation, to achieve the effect of treating both the root cause and symptoms; meanwhile, we also provided Chinese medicine dietary guidance to the patients, promoting their physical recovery. Through the combination of these TCM featured care and individualized care, we aimed to provide more comprehensive and meticulous nursing services for patients with upper limb fractures, promoting their recovery process.

### 2.4. Observation indicators

Record the time for swelling regression, time for pain relief, and length of hospital stay to evaluate the symptom recovery time of the patients.

Use the Fugl-Meyer Assessment Upper Extremity (FMA-UE) scale to assess the recovery of upper limb function before and after nursing care.

Use the Hamilton Anxiety Scale (HAMA) to assess the degree of anxiety before and after nursing care, and understand the changes in negative emotions.

Use the Short Form Health Survey (SF-36) to assess the quality of life before and after nursing care, including dimensions such as energy, overall health, and emotional function.

### 2.5. Statistical analysis

All statistical analyses were performed using SPSS version 30.0 (IBM Corp., Armonk). Continuous variables were first assessed for normality using the Shapiro–Wilk test (*α* = 0.05). Homogeneity of variance was verified through Levene test (*α* = 0.10). For normally distributed data with equal variances, independent samples *t* tests were used for between-group comparisons. Measurement data are expressed as (x±s), and independent sample *t* tests were used for intergroup comparisons; count data are expressed as (n [%]) and chi-square tests were used for intergroup comparisons, with *P* < .05 indicating statistical significance.

## 3. Results

Based on the data analysis from Tables 1–3, the following conclusions can be drawn:

**Table 1:** Comparison of symptom recovery time between 2 groups of patients.

Group	N	Time for pain relief (d)	Time for edema resolution (d)	Length of hospital stay (d)
Control group	90	8.62 ± 2.55	7.81 ± 2.09	10.81 ± 2.48
Observation group	90	7.18 ± 1.43	6.67 ± 1.12	9.27 ± 2.10
*t* value		3.291	3.162	3.153
*P* value		.002	.003	.002

**Table 2:** FMA-UE score comparison between groups.

Group	N	Pre-nursing FMA-UE score (points)	Post-nursing FMA-UE score (points)	*t* value	*P* value
Control group	90	29.03 ± 5.54	41.72 ± 4.79	11.605	<.001
Observation group	90	28.76 ± 5.47	50.51 ± 3.25	22.894	<.001
*t* value		0.251	10.213		
*P* value		.814	<.001		

FMA-UE = Fugl-Meyer Assessment Upper Extremity.

**Table 3:** Comparison of HAMA and SF-36 scores between groups.

Group	N	HAMA score (before nursing)	HAMA score (after nursing)	SF-36 score (before nursing)	SF-36 score (after nursing)
Control group	90	13.62 ± 3.63	8.59 ± 2.54	596.31 ± 25.89	690.18 ± 51.35
Observation group	90	13.29 ± 3.26	5.47 ± 1.87	588.02 ± 34.09	714.15 ± 20.87
*t* value		0.456	6.605	1.157	2.921
*P* value		.651	<.001	.252	.004

HAMA = Hamilton Anxiety Scale, SF-36 = Short Form Health Survey.

The clinical data of the patients were retrospectively analyzed, and based on different nursing methods, they were divided into control group and observation group, with 90 cases in each group. In the control group, there were 56 males and 34 females, with an age range of 22 to 62 years and a mean age of (47.31 ± 6.12) years. The education levels included 44 cases with junior high school or below, 30 cases with high school or technical secondary school, and 16 cases with college or above. The types of fractures included 6 cases of metacarpal fractures, 12 cases of clavicle fractures, 26 cases of radius and ulna shaft fractures, 32 cases of humerus shaft fractures, 4 cases of scapular fractures, and 10 other cases. In the observation group, there were 50 males and 40 females, with an age range of 24 to 64 years and a mean age of (46.69 ± 6.53) years. The education levels included 50 cases with junior high school or below, 26 cases with high school or technical secondary school, and 14 cases with college or above. The types of fractures included 10 cases of metacarpal fractures, 10 cases of clavicle shaft fractures, 22 cases of radius and ulna shaft fractures, 26 cases of humerus shaft fractures, 8 cases of scapular fractures, and 14 other cases. The comparison of general data between the 2 groups showed no statistically significant differences (*P* > .05), indicating comparability.

### 3.1. Comparison of symptom recovery time

In terms of symptom recovery time, the observation group had shorter times for pain relief, swelling regression, and length of hospital stay compared to the control group. Specifically, the pain relief time in the observation group was 7.18 ± 1.43 days, swelling regression time was 6.67 ± 1.12 days, and length of hospital stay was 9.27 ± 2.10 days; while in the control group, the corresponding times were 8.62 ± 2.55 days, 7.81 ± 2.09 days, and 10.81 ± 2.48 days, respectively. The *t* test showed that the differences between the 2 groups in pain relief time, swelling regression time, and length of hospital stay were statistically significant (*P* < .005). This indicates that through the combination of TCM featured care and individualized care, patients experienced a significant reduction in symptom recovery time.

### 3.2. Comparison of upper limb function scores

In terms of FMA-UE scores, the 2 groups of patients had similar FMA-UE scores before nursing care, with no statistically significant difference (*P* > .05). However, after nursing care, the FMA-UE scores of the observation group were significantly higher than those of the control group, and the difference was statistically significant (*P* < .001). This suggests that TCM featured care combined with individualized care achieved significant improvement in upper limb motor function for patients.

### 3.3. Comparison of negative emotions and quality of life scores

In terms of HAMA and SF-36 scores, we found that before nursing care, the HAMA scores of the 2 groups of patients were similar, with no statistically significant difference (*P* > .05), and the SF-36 scores were also at similar levels. After nursing care, the HAMA scores of the observation group significantly decreased, with a greater reduction than that of the control group, and the difference was statistically significant (*P* < .001). Similarly, the SF-36 scores of the observation group also significantly improved, with a greater increase than that of the control group, and the difference was also statistically significant (*P* < .05). This indicates that TCM featured care combined with individualized care played a positive role in reducing patient anxiety and improving quality of life.

## 4. Discussion

With the continuous development of modern medicine, the treatment of fracture patients has evolved beyond mere physiological care to encompass their psychological, social, and other multifaceted needs.^[15,16]^ Especially in cases of upper limb fractures, the degree of functional recovery directly impacts the quality of life of patients.^[17]^ Therefore, exploring how to better provide nursing care for upper limb fracture patients is of paramount practical significance. This study aimed to provide more scientific and effective guidance for clinical nursing by comparing the application effects of TCM featured care combined with individualized care and conventional care in upper limb fracture patients.

Firstly, from the perspective of symptom recovery time, TCM featured care combined with individualized care demonstrated significant advantages. This is mainly attributed to the core concepts of TCM care, namely holistic view and syndrome differentiation and treatment.^[18–21]^ TCM care emphasizes the overall condition of patients, formulating personalized nursing plans through the 4 diagnostic methods of observation, listening, questioning, and pulse feeling, comprehensively understanding the patient’s condition. Moreover, TCM care emphasizes the concept of “preventing disease before it occurs,” taking proactive preventive measures before the disease develops further.^[22,23]^ This holistic and preventive nursing concept makes TCM featured care more advantageous in promoting patient symptom recovery. Additionally, the introduction of individualized care makes nursing work more tailored to the actual needs of patients, enhancing the specificity and effectiveness of care.

Secondly, in terms of upper limb function recovery, TCM featured care combined with individualized care also achieved significant results.^[24–26]^ TCM nursing techniques such as massage, acupuncture, and moxibustion can effectively promote local blood circulation, accelerate the absorption and regression of inflammation, thereby facilitating swelling reduction and pain relief. Moreover, TCM care emphasizes functional exercises for patients, promoting the recovery of upper limb function. Individualized care tailors rehabilitation plans to the specific conditions of patients, ensuring that patients undergo rehabilitation training in the best possible state. This comprehensive nursing model has led to better recovery of upper limb function in patients.

Furthermore, from the perspectives of negative emotions and quality of life, TCM featured care combined with individualized care also demonstrated significant advantages. Due to pain, restricted activity, and other reasons, fracture patients often experience anxiety, depression, and other negative emotions, which subsequently affect their quality of life.^[27]^ TCM featured care provides psychological counseling, emotional regulation, and other methods to help patients alleviate negative emotions and build confidence in overcoming the disease.^[28,29]^ Additionally, TCM care also emphasizes dietary regulation and lifestyle care to improve the quality of life of patients. Individualized care focuses more on the personalized needs of patients, providing tailored psychological and social support to help patients better cope with life challenges. This comprehensive nursing model effectively alleviates negative emotions and improves the quality of life of patients.

Moreover, it is worth noting that the implementation of TCM featured care combined with individualized care requires certain professional knowledge and skills. Nursing staff need to master the basic theories and techniques of TCM, as well as possess rich clinical nursing experience and keen observational skills to formulate personalized nursing plans based on the specific conditions of patients. Therefore, in practical application, it is necessary to strengthen the training and education of nursing staff, improve their professional competence and skill levels, and ensure the effective implementation of TCM featured care combined with individualized care.

While this study demonstrates promising results, several limitations should be acknowledged. First, the single-center design (Wuhan Fourth Hospital) and homogeneous sample (Chinese Han population) may limit generalizability to other ethnic groups or healthcare settings. Regional treatment preferences and genetic factors could influence outcomes, potentially introducing selection bias. Second, our 3-month follow-up period focused on short-term recovery metrics but did not evaluate long-term functional outcomes (>6 months) or potential late complications such as posttraumatic arthritis or chronic pain syndromes. Third, the retrospective nature of data collection may have introduced recall bias, despite our rigorous data verification processes. Finally, the non-blinded design (inherent in nursing intervention studies) could have influenced both patient-reported outcomes and caregiver assessments.

To address the current study’s limitations and further explore the long-term benefits of the integrated care model, future research designs should focus on the following coordinated directions: First, multicenter randomized controlled trials spanning diverse healthcare systems and ethnic groups should be conducted, extending follow-up periods beyond 12 months. These trials should employ standardized radiographic union scales (e.g., Upper Limb Radiographic Union Score) combined with patient-reported outcomes (e.g., PROMIS® Upper Extremity Short Form) for comprehensive evaluation. Second, mechanistic investigations should be incorporated, integrating dynamic contrast-enhanced magnetic resonance imaging to assess regional microcirculatory changes, while simultaneously monitoring dynamic expression of key fracture healing biomarkers such as vascular endothelial growth factor and transforming growth factor-β1 to quantify the biological effects of TCM interventions. Concurrently, stratified study cohorts should be established to develop adaptive intervention protocols for fracture patients with comorbidities like diabetes and osteoporosis. Furthermore, health economic evaluations should be implemented using quality-adjusted life years and incremental cost-effectiveness ratios to compare long-term cost-effectiveness between this model and conventional rehabilitation pathways. Through this multidimensional research framework, we can systematically validate the universality, mechanistic basis, and health economic value of the combined care model, ultimately providing high-level evidence to support clinical translation.

This study proposes a practical framework for integrating TCM into multidisciplinary fracture rehabilitation: Establish TCM certification for physiotherapists focusing on meridian massage, analgesic acupoints (LI4/SJ5), and moxibustion osteogenesis techniques; Develop tripartite care models (nurses/physiotherapists/TCM practitioners) combining biomechanical assessments with “tendon-bone simultaneity” theory through Baduanjin-incorporated rehabilitation protocols; Create cross-cultural adaptation kits (Dietary Approaches to Stop Hypertension-TCM syndrome hybrid scales with EU regulatory guidelines). Future priorities include: dose-response studies of integrated interventions, cost-effectiveness analysis of cross-training, and safety evaluations of combined manual therapies to facilitate evidence-based translation.

In summary, TCM featured care combined with individualized care demonstrates significant advantages in nursing care for upper limb fracture patients, effectively promoting symptom recovery, improving upper limb function, alleviating negative emotions, and enhancing quality of life. This nursing model not only conforms to the holistic and individualized development trend of modern medicine but also embodies the unique advantages and value of TCM care. Therefore, we recommend further promoting and applying the model of TCM featured care combined with individualized care in clinical practice to provide more comprehensive and effective nursing services for upper limb fracture patients. Meanwhile, continuous summarization of experiences and lessons, as well as refinement of nursing plans and techniques, are necessary to better meet the needs of patients and improve nursing quality.

## Author contributions

**Conceptualization:** Shuman Qian, Ying Ma.

**Data curation:** Shuman Qian, Ying Ma.

**Formal analysis:** Shuman Qian, Ying Ma.

**Investigation:** Shuman Qian, Ying Ma.

**Methodology:** Shuman Qian, Ying Ma.

**Writing – original draft:** Shuman Qian, Ying Ma.

**Writing – review & editing:** Shuman Qian, Ying Ma.
